# Effectiveness of a Lifestyle Intervention in Patients with Type 2 Diabetes: The Physical Activity and Nutrition for Diabetes in Alberta (PANDA) Trial

**DOI:** 10.3390/healthcare4040073

**Published:** 2016-09-27

**Authors:** Ghada Asaad, Diana C. Soria-Contreras, Rhonda C. Bell, Catherine B. Chan

**Affiliations:** 1 Department of Agricultural, Food and Nutritional Science, University of Alberta, Edmonton, AB T6G 2R3, Canada; gasaad@ualberta.ca (G.A.); soria@ualberta.ca (D.C.S.-C.); rhonda.bell@ualberta.ca (R.C.B.); 2 Department of Physiology, University of Alberta, Edmonton, AB T6G 2R3, Canada; 3 Diabetes, Obesity and Nutrition Strategic Clinical Network, Alberta Health Services, Edmonton, AB T6G 2R3, Canada

**Keywords:** type 2 diabetes, intervention, menu plan, glycemic control, dietary adherence, diet quality

## Abstract

Type 2 diabetes (T2D) patients often find integrating a new dietary pattern into their lifestyle challenging; therefore, the PANDA (Physical Activity and Nutrition for Diabetes in Alberta) menu plan intervention was developed to help people incorporate the Canadian Diabetes Association (CDA) nutrition therapy guidelines into their daily lives. The menu plan focused on recipes and foods that were accessible, available and acceptable to Albertans. The objective was to evaluate the effectiveness of the intervention on blood glucose control and dietary adherence and quality among patients with T2D. Participants with T2D (*n* = 73) enrolled in a single-arm incorporating interactive education based on a four-week menu plan that incorporated the recommendations of the CDA nutrition therapy guidelines. Post-intervention follow-up was conducted at three and six months. After three months, there were beneficial changes in A1c (−0.7%), body mass index (BMI, −0.6 kg/m^2^), diastolic blood pressure (−4 mmHg), total cholesterol (−63 mg/dL), HDL- (+28 mg/dL) and LDL-cholesterol (−89 mg/dL), Healthy Eating Index (+2.1 score) and perceived dietary adherence (+8.5 score) (all *p* < 0.05). The significant improvements in A1c, BMI and lipids were maintained at six months. The PANDA menu plan intervention was effective in improving glycemic control and diet quality. The results suggest that a dietary intervention incorporating interactive education sessions focused on menu planning with familiar, accessible foods may be effective for diabetes management.

## 1. Introduction

Diabetes is a major global health issue with over 0.5 billion individuals projected to be diagnosed by 2030 [1]. In Canada, by 2019–2020 the number is expected to reach 3.7 million, approximately 10% of the population [2] with an estimated cost of $16.9 billion [3] to the Canadian health care system. The Canadian Diabetes Association (CDA) Clinical Practice Guidelines (CPG) provide evidence-based recommendations for nutrition therapy as part of effective diabetes management [4]. Nutrition therapy can reduce glycated hemoglobin (A1c) by 1%–2%, improve serum cholesterol levels and facilitate weight management [4]. Despite these benefits, diabetic patients find it difficult to integrate a dietary pattern consistent with the recommendations into their lifestyle [5,6]. Thus, not surprisingly, type 2 diabetes (T2D) patients have poor adherence to dietary recommendations [7,8]. Personal factors that may be barriers to adherence include language and communication skills, lack of knowledge or motivation, taste preferences and cravings, cooking skills, and lack of family and social support [9,10]. Acculturation, lack of cultural acceptability of recommended diets and the cost of recommended foods are also barriers to diabetic diet adherence [10,11]. Diabetes educators recognize that clients’ ability to incorporate recommendations is affected by these factors but that many clients may not have strategies and tools to overcome these barriers [12]. People with T2D identified ongoing professional and peer support and multi-level programming as potential solutions to address barriers to behavior change [12].

Environmental barriers also affect dietary adherence. The 4-A Framework, derived from the food security literature [13], suggests that foods recommended in nutrition programs should be adequate, accessible, acceptable and available. Adequacy means the diet meets guidelines that lead to better primary (blood glucose control) and secondary outcomes (reduce complications). Accessible refers to financial and physical accessibility of foods. Foods must be acceptable from multiple perspectives: hedonic qualities, culture, traditions and usual consumption habits. Finally, foods must be generally available to the consumer population of interest, e.g., locally grown or regularly imported [11].

Another challenge for T2D patients is translating nutrition recommendations into concrete operational plans such as food procurement, recipe selection, managing time to include food preparation, and budgeting [14]. Menu plan and grocery list interventions were effective strategies for weight control as well as diabetes management [15,16] but did not incorporate elements of the 4-A Framework. However, addressing environmental barriers may facilitate patient adoption of and adherence to dietary recommendations. To address this, a four-week menu plan based on the principles of the 4-A Framework was developed [17] to meet the CDA nutrition therapy guidelines [4]. A phase 1 pilot-test of 15 participants conducted to test its feasibility and efficacy to improve diabetes outcomes found reductions (*p* < 0.05) in A1c (−1%), weight (−2.6 kg) and improvement in HDL-cholesterol (HDL-C) (+0.2 mmol/L) after three months [17]. Focus group interviews conducted to qualitatively assess facilitators and barriers to implementing the menu plan showed that the menu plan was acceptable and useful for the participants [17,18]. Hence, results of the pilot study justified a larger trial, this time incorporating a structured education program with multiple opportunities for skill-building and increasing knowledge, as well as peer support. The objective of this study was to evaluate the effectiveness of the menu plan plus education sessions among people with T2D in improving glycemic control and promoting dietary changes.

## 2. Materials and Methods

### 2.1. Participants

**Figure 1:**
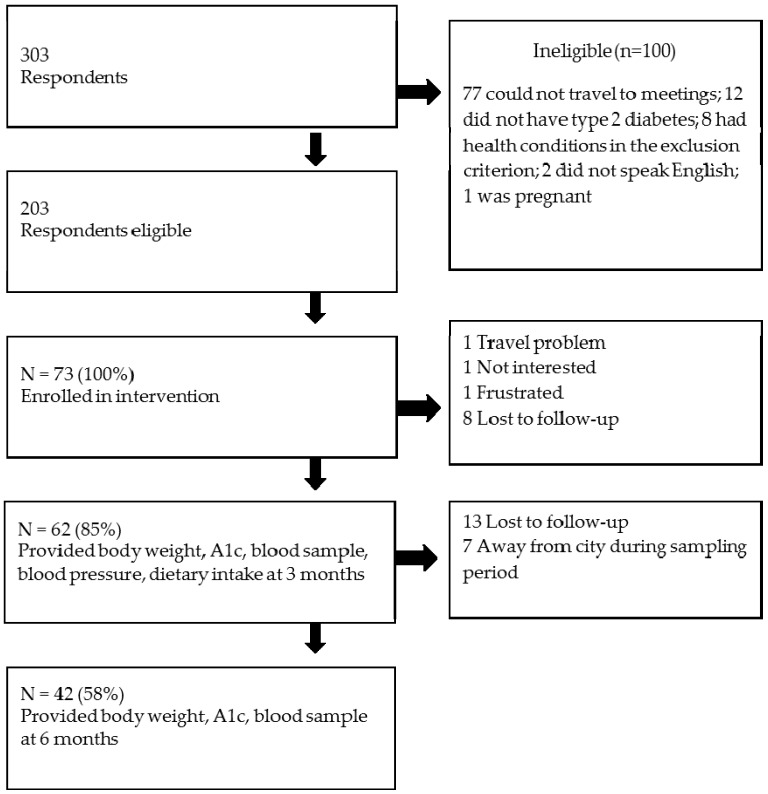
Participant recruitment and retention flow chart.

Participants were recruited for this study using posters hung in public places at the University of Alberta in Edmonton, word-of-mouth, email invitations sent to a contact list of potential participants maintained by the Alberta Diabetes Institute, and publicity by local media. There were 303 respondents who expressed interest in participating in this study (Figure 1), of whom 203 were deemed eligible based on a brief telephone interview. A personal screening interview was conducted to obtain demographic and baseline information. Respondents met the inclusion criteria if they self-identified as having T2D, could speak and write English, and had attended an Alberta Health Services-conducted diabetes education session. The exclusion criteria were: concomitant diseases or conditions that would preclude them following the menu plan, or having type 1 diabetes, severe diabetes complications such as kidney failure or being pregnant. The selection criteria were: subjects who met the inclusion criteria, were able to commit time to the study and able and willing to travel to weekly meetings (on Friday or Saturday) at the University of Alberta campus in Edmonton, Alberta, Canada. From eligible respondents, an approximately equal number of males and females, as well as variation in A1c (where known), age, and ethnicity of participants was selected. In the event that more than 1 respondent met the selection criteria, the date of response was considered, with the earlier date preferred. Selection ceased once the required number of subjects was reached. An initial sample size of 51 participants was calculated (alpha = 0.05, 1 − beta = 0.8, paired *t*-test) with the aim of detecting a 0.5% change in A1c, which was selected to be clinically relevant. Assuming a drop-out rate of 30% based on the pilot study [17], 73 participants were enrolled into 5 small groups, with 12–14 people/group. To minimize drop out, the study coordinator used different strategies such as: following up with participants by phone and email, motivating participants (e.g., setting a goal to achieve, active learning activities), reducing the travel burden by providing complimentary parking in a nearby lot or paying for transit, keeping the sessions short, and allowing participants to choose either a weekend or weekday intervention timeslot. The study was conducted in the Human Nutrition Research Unit; all participants provided written, informed consent prior to starting the study. The protocol for this study was approved by the University of Alberta Research Ethics Board (ClinicalTrials.gov registration NCT01625507).

### 2.2. Study Design, Assessments, and Endpoints

This study was a single-arm, pre-post intervention study entitled Physical Activity and Nutrition for Diabetes in Alberta (PANDA)-Nutrition Arm. The single-arm pre- and post-test design was use to evaluate the effect of explicit comparisons of the intervention. Participants completed assessments of dietary intake, physical activity, Diabetes Self-Efficacy Scale (DSES) [19], and metabolic and anthropometric/health characteristics at baseline (following recruitment to the study and before the first educational meeting), within two weeks following completion of the intervention (three months) and six months following enrolment. Current dietary intake was assessed using three 24-h dietary recalls (2 weekdays and 1 weekend day) through a web-based questionnaire (Webspan; [20]). Nutrient intake was determined by linking the food intake data to the Canadian Nutrient File [21] after the food intake data had been carefully reviewed and cleaned to remove duplicate or implausible entries. Implausible total energy was considered to be outside the range of 500–3500 kcal/day for women and 800–4000 kcal/day for men [22]; however, none of the participants reported implausible total energy. Estimated energy requirement (EER) for each participant was calculated by using the Institute of Medicine method [23] with physical activity level estimated by converting steps/day from pedometer readings (see below) to categories from sedentary (<5000 steps/day) to highly active (≥12,500 steps/day) [24]. The Goldberg cut-off was used to identify under-reported energy intake [25]. Diet quality was assessed by calculating the Healthy Eating Index (HEI) adapted to the Canadian population [26]. Participants’ perceptions of their dietary adherence to CDA Nutrition Therapy Guidelines were assessed by the Perceived Dietary Adherence Questionnaire (PDAQ) [27]. Prior to and post-intervention, participants were asked to report all the medications that they used (name of drug, frequency of use and dose) as a potential confounder of intervention outcomes.

Physical activity was assessed by a pedometer for three consecutive days. Instructions regarding use of the pedometer were given by the research coordinator at the time of the first meeting, and a sealed pedometer was provided to each participant at the first and seventh meetings. The pedometer was attached to the belt or waistband of the participant’s clothing. Participants wore the pedometers from the time of rising in the morning until bedtime during the monitoring period. Pedometers were removed during water-related activities (e.g., swimming, showering). After 3 days, participants were instructed to remove the seal and to email the study coordinator the total number of steps. The pedometers were retrieved from the participants at the second and eighth meeting. The DSES was used to measure participants’ perceived confidence in performing self-care activities related to nutrition, exercise, glucose control and diabetes-related decision-making [19]. Changes in the DSES score between baseline and post-program assessment were used to assess potential influences in successful behavioral change.

Glycemic control was assessed using a finger prick blood sample (DCA 2000þ Analyzer; Bayer, Tarrytown, NY, USA). Fasting (minimum 12 h since last meal or snack) venous blood samples were collected to assess triglycerides (TG), total-C, LDL-C and HDL-C. Blood samples were centrifuged (3500 rpm), and serum was removed and frozen at −80 °C until analyzed using enzymatic colorimetric assays (Wako Chemicals, Richmond, VA, USA) for each metabolite except LDL-C levels were determined by using the subtraction method.

Body weight was measured to the nearest 0.1 kg with the participant wearing light indoor clothing and without shoes using a digital scale (Health-o-Meter Professional Series; Sunbeam, Boca Raton, FL, USA), height was measured to the nearest 0.1 cm (Heightronic Digital Stadiometer; QuickMedical, Northbend, WA, USA) and body mass index (BMI) was calculated from height and weight measures. Waist circumference was measured to the nearest 0.1 cm with the participant in a standing position, with a non-stretch tape place midway between the lateral lower ribs and the iliac crests after a moderate expiration. Body composition was measured using air displacement plethysmography (Bod Pod; COSMED USA, Concord, CA, USA). Blood pressure was determined following a 5-min rest period with the participant seated by an auto-inflated digital unit (UA-767CN; LifeSource, Japan). Blood pressure was measured 3 times, each 2 minutes apart, and the results averaged. All measurements were taken by trained personnel following standardized procedures.

### 2.3. Intervention

**Figure 2:**
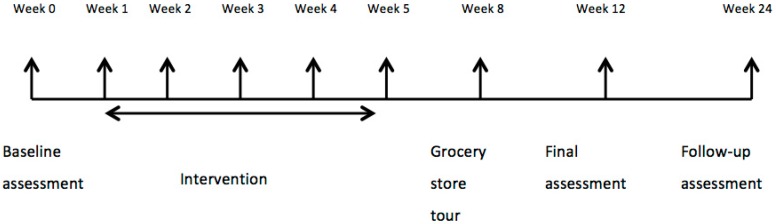
Intervention timeline.

In accordance with best practices in nutrition interventions for diabetic patients, this study used Social Cognitive Theory as a theoretical model to guide the overall behavior change intervention. This model emphasizes skill acquisition through practice with feedback, support and positive reinforcement [28], goal-setting, self-monitoring and problem-solving as behavior change strategies. Weekly meetings approximately 1.5–2 h in length were conducted by a facilitator with a M.Sc. in human nutrition and trained as a dietician. The study held at the Human Nutrition Research Unit in the Alberta Diabetes Institute, the University of Alberta. The intervention curriculum consisted of five sessions (Figure 2, weeks 1–5): the first week focused on Canada’s Food Guide (CFG) food groups, serving sizes and number of recommended servings/day an open-ended discussion of facilitators and barriers to adhering to a dietary pattern consistent with CFG. Participants were encouraged to set individualized dietary goals based on review of their personal baseline dietary intake data (provided during the session) of food groups and servings. The second through fifth weeks started with a discussion of participants’ experiences and reflections on attaining dietary goals and factors that contributed to their dietary behaviors since the last session. At the end of each session, participants set new goals for the upcoming week. In week 2 of the curriculum, the menu plan was introduced. The participants were provided with, and based discussions on, a nutritionally adequate 4-week menu that incorporated suggested foods and ingredients that were locally available, financially and physically accessible, culturally acceptable and met the serving recommendations of CFG [29]. Additional information on the menu plan is available at www.pureprairie.ca and a sample menu is provided in Supplementary Materials Table S1. Week 3 included activities demonstrating how the menu plan could be adapted for energy needs, family size and cultural preferences, and included a cooking demonstration. In week 4, participants discussed food choices when dining out and practiced reading food labels. In week 5, participants were provided with information and had opportunity to practice carbohydrate counting and choosing carbohydrate-rich foods with a low glycemic index. In week 8 participants toured a grocery store with a dietitian. In the week prior to the post-intervention assessment (i.e., at 11 weeks), participants completed three 24-h recalls. All participants were given a $50 gift card for a grocery store of their choice for taking part in the study, and were reimbursed for parking or public transit expenses incurred. Finally, participants were invited to a post-intervention assessment 6 months after entry into the study to measure the longer-term effects of the PANDA program on biological outcomes.

### 2.4. Statistical Analysis

Baseline data are reported as mean ± SD or proportions as appropriate. Baseline differences between males and females were determined using a chi-square test for categorical variables or unpaired *t*-test for continuous variables with *p* < 0.05 considered significant. Outcome analyses were performed for those with complete data at baseline and post-intervention using intention-to-treat analysis conducted with 5 sets of multiple imputations (Amelia II package in R statistical software). The multiple imputations generate values based on the expectation–maximization algorithm [30]. Imputation at 6 months was based on data obtained at baseline and 3 months. Differences between baseline and post-intervention measures were assessed and are reported as the mean difference between baseline and post intervention values with 95% confidence intervals (CI). Pre-post differences were assessed by paired *t*-test. Sensitivity analysis was carried out to estimate to what the extent under-reporting of energy intakes by participants affected nutritional intake outcomes and effects on A1c by comparing those with under-reported versus acceptable energy intakes relative to EER. Pearson correlations were computed with all variables versus primary outcomes (A1c, HEI) to identify potential determinants for linear regression modeling. Variable selection in the linear regression models was based on literature review and bivariate correlation (*p* < 0.2). Multiple regression analyses were used to examine the relationship between changes in BMI, HDL-C, total calories, HEI and PDAQ scores with post-intervention changes in A1c. This model was adjusted for potential confounding by age, gender, baseline physical activity, baseline HEI, baseline BMI and baseline A1c. Similarly, multiple linear regression was used to examine the relationship between post-intervention changes in HEI score and changes in total calories, fat intake, saturated fat intake, added sugar intake and sodium intake as well as behavioral indicators, PDAQ and DSES. This model was adjusted for age, gender baseline A1c, and HEI baseline. Data are reported as the change in A1c (unstandardized coefficient, B with 95% CI) predicted by a set change in the variable of interest. These analyses were conducted using SPSS (IBM, version 22, IBM Analytics, Armonk, NY, USA); a *p*-value of < 0.05 was considered statistically significant.

## 3. Results

### 3.1. Study Participants at Baseline

Participants were older adults and had been diagnosed with T2D for approximately nine years. Similar numbers of men (*n* = 39) and women (*n* = 34) participated in this study (Figure 1 and Table 1). Eighty-five percent of participants completed the intervention and all assessments at three months, and 58% of participant returned for the assessments at six months post intervention. Men and women in this study had similar demographic and health-related characteristics except that men had been diagnosed with diabetes for a longer period of time, scored higher than women on the DSES, and had higher systolic blood pressure. The majority of participants (74%) reported taking oral medication to control hyperglycemia. Hypertension (57.5%) and dyslipidemia (47.9%) medications were most frequently reported as additional medications by participants.

At baseline, energy intake was ~2100 kcal/day but was underestimated by two-thirds of participants (Table 1). The CDA-recommended macronutrient distribution ranges for carbohydrate and protein were met by the participants, but saturated fat (12% total energy) exceeded the recommended contribution to total energy (7%) and total fat was slightly higher than the recommended range (36% versus 35%). Added sugar (50 g/day) was also in the acceptable range (<10% of total energy) but fiber (22 g/day) was less than recommended (25 g/day) and sodium intake (3.4 g/day) exceeded the tolerable upper limit of 2.3 g/day. Generally, HEI scores indicated that participants “need improvement” although two participants had poor diet quality (score <51) and eight had good diet quality (score >80). HEI scores correlated significantly with PDAQ scores (*r* = 0.418, *p* < 0.001).

### 3.2. Effect of PANDA–Nutrition Arm on A1c and Secondary Biological Outcomes

Biological outcomes for the cohort are reported in Table 2. Three months after the initiation of the PANDA intervention, A1c had decreased by 0.7% (95% CI 0.4% to 1.0%). Secondary outcomes with significant improvements included: waist circumference, BMI, fat mass (kg), fat free mass (%), systolic and diastolic blood pressure, total-C, HDL-C and LDL-C. Physical activity was increased. At six-month follow-up, significant reductions in A1c, waist circumference, BMI, total-C, and LDL-C were still detected along with increased HDL-C (Table 2). No changes in hyperglycemia, hypertension and dyslipidemia medications were reported by participants after the PANDA intervention. However, some participants (13.7%) did not report the frequency or dose of medications used.

Sensitivity analysis to determine the impact of under-reporting of energy intake on the primary outcome was conducted. A1c decreased by 0.6% (95% CI 0.3, 1.0) in individuals with under-reported energy compared with 1.1% (95% CI 0.5, 1.8) in those with acceptable reporting, which was not statistically different.

### 3.3. Effect of PANDA—Nutrition Arm on Dietary Adherence and Diet Quality at Three Months

Changes in dietary adherence were measured by comparing pre- and post-intervention, averaged 24-h dietary recall data (Table 3). There were post-intervention reductions in intakes of total energy, and total fat, protein, added sugar, saturated fat, sodium, and sodium density (mg sodium/1000 kcal). Macronutrient distribution did not change significantly. HEI improved by +2.1 points (95% CI 0.2 to 4.1). There was a significant positive shift in the number of participants in HEI category after the intervention (X^2^ (*n* = 73) = 29.31, *p* < 0.001). Seven participants whose diet quality was categorized as “needs improvement” prior to the intervention improved to “good” diet quality. After 3 months, PDAQ score significantly increased by 8.5 points as did the score on the Diabetes Self-Efficacy scale by +0.7 (Table 2).

Changes in nutrient intake in the subsample of under-reporters of energy at baseline (*n* = 49) compared with acceptable reporters (*n* = 18) were assessed. Under- versus acceptable-reporters had the following changes (95% CI) in nutrient parameters: energy −167 kcal (−332, −2) versus −301 kcal (−509, −92); total fat −7.8 g (−17.2, 1.5) versus −21.6 g (−36.2, −7.0), protein −5.9 g (−12.6, 0.8) versus −5.6 g (−17.4, 6.2), added sugar −10.8 g (−22.8, 1.2) versus −5.1 g (−13.2, −3.1), sodium −0.42 g (−0.76, −0.07) versus −1.2 g (−1.8, −0.06). Only the reduction in sodium was different between the groups (*p* < 0.001). HEI score changes were 1.3 (−0.8, 3.4) versus 4.4 (−0.6, 9.4) in under- versus acceptable-reporters, respectively. Notably, the number of participants acceptably reporting energy intake instead of under-reporting was increased by 12 individuals.

### 3.4. Predictors of Changes in A1c and HEI Score

Multiple linear regression analysis was carried out to examine the relationship between changes in A1c relative to changes in nutritional variables (total calories and HEI), biological variables (BMI, HDL-C) and physical activity at three months (Table 4). In the unadjusted model (Model 1), an increase in HDL-C and physical activity predicted reductions in A1c. In Model 2, adjusting for baseline A1c, age and gender somewhat attenuated HDL-C as a predictor of reductions in A1c and physical activity became non-significant. However, the adjustment strengthened the relationship of BMI with reduced A1c. In both models, change in HEI score was not significant (*p* > 0.1). To examine influences on changes in HEI score, multiple linear regression was carried out including nutritional changes identified as predictors (Table 5). In both unadjusted (B = −0.111 (95% CI −0.186, −0.035)) and adjusted models (B = −0.117 (95% CI −0.195, −0.039)), a decrease in saturated fat intake was the only significant variable associated with increased HEI. Neither changes in PDAQ nor DSES score were associated with change in HEI in simple linear regressions and so were not included in the model.

## 4. Discussion

The results of this study indicate that the PANDA Nutrition Arm effectively improved clinical outcomes and dietary adherence in T2D participants. The menu plan was based on the 4-A Framework for content and the intervention education utilized Social Cognitive Theory for process. Significant improvements were found across A1c, anthropometric variables and lipid profile variables after three months and sustained at six months. Effectiveness and sustainability of the menu plan program to improve clinical outcomes occurred despite the fact that the intervention focused on nutrition education and healthy eating patterns, not weight loss.

We observed important changes in participants’ eating patterns following the PANDA program. Diet quality (HEI score) improved modestly after three months. The improved total score was attributable to increased whole fruit intake along with decreased saturated fat and sodium sub-scores. A meta-analysis of 15 cohort studies reported that diet quality was associated with reduced risk of all-cause mortality, CVD, cancer and T2D [31]. Therefore, improvement of diet quality may have positive consequences in the risk of further complications for people with T2D. Although only a modest improvement in HEI score was recorded, reductions in total energy, total fat, and sodium intake were achieved. Total energy is important in terms of glycemic control. Some studies have shown improved in glycemic control when total energy is restricted [32,33]. Fat intake may also affect glycemic control. A low-fat calorie-restricted diet can improve glycemic control among in T2D patients [34]. The HEI score does not take into account caloric reduction or all aspects of a diabetes diet, for example recommendations to consume low glycemic index foods and may underestimate dietary changes in this population.

The biggest change in diet was a reduction in sodium intake that persisted after adjusting for energy intake. This improved sodium consumption pattern may reflect changes in eating habits of study participants, for example eating more food cooked from scratch vs. restaurant meals [35]. A study of the DASH diet showed that limiting sodium intake to <2300 mg/day predicted reduced blood pressure [36]. Elsewhere, a 24-week meal preparation intervention was conducted in T2D patients. The program successfully reduced weight, A1c, and there was a trend toward lower blood pressure but sodium intake was not documented [37]. Our study showed a significant reduction in systolic blood pressure by 4.1 mm Hg and diastolic blood pressure by 1.7 mm Hg. Therefore, the PANDA intervention menu plan led to overall improved diet quality, which may have contributed to improved blood pressure control.

Adherence to the Canadian nutrition recommendations for diabetes was assessed by the PDAQ. We found a significant improvement in the PDAQ score after three months (+8.5 points), which implies that the PANDA Nutrition Arm is feasible for helping people with diabetes to follow the dietary recommendations, and possibly more sensitive to behavioral change than assessment of dietary intake or the HEI score because it asked specific questions about diabetes nutrition recommendations, fiber intake and low glycemic foods for example.

The PANDA Nutrition Arm led to a significant improvement in A1c at three months (−0.7%) that was sustained six months after the program. This change in A1c is greater than found in several other nutrition interventions [16,38]. The change in A1c, although modest, is considered clinically relevant and can reduce the risk of long-term diabetes complications [39,40]. However, it is not clear whether improvement in A1c was related to diet or physical activity changes since neither emerged as a strong predictor in the linear regression models and both can lead to weight loss [41]. In our study, weight loss was the strongest predictor of improved A1c, with −1 kg/m^2^ predicting a 0.1% reduction in A1c. A confounder was revealed through categorization of participants as acceptable or under-reporters of energy intake because the number of acceptable reporters increased from 18 to 30 from baseline to three months. Therefore, changes in energy intake and other nutrient variables were underestimated, which may have contributed to lack of significance in the models. Our results are consistent with the Look AHEAD cohort, which showed that a modest weight loss of 2%–5% was associated with significant improvement in A1c (1.80% (95% CI 1.44–2.24)), and other CVD risk factors [42]. We also observed significant changes in fat mass and waist circumference similar in magnitude to those observed in the Look AHEAD trial [43]. The inverse relationship between changes in A1c and serum HDL-C shown in the PANDA intervention has been reported by others [44,45] but is not as well established as that between A1c and BMI and is not seen in all studies [46,47]. However, a prospective trial demonstrated that low baseline HDL-C predicted more rapid long-term progression of T2D [46]. HDL-C promotes insulin secretion and protection from apoptosis of beta-cells [48], which may be related to HDL-C-mediated anti-oxidative and cellular cholesterol efflux activities [49]. HDL-C also improves insulin-independent glucose uptake into skeletal muscle [50]. Thus, some authors recommend that increasing HDL-C be considered an important strategy to improve glycemic control [51].

Cardiovascular disease (CVD) is the leading cause of death among diabetic patients. Lifestyle interventions have the potential to improve several risk factors for CVD such as glycemic control, blood pressure and lipids [4,17,52]. After the PANDA program, we observed improvements in total-C, HDL-C and LDL-C at three and six months. Moreover, consistent with our pilot study [17], a significant reduction in blood pressure was found. The significant changes in clinical outcomes documented after the PANDA intervention, if sustained over the long-term, may lower risk of future CVD.

One major difference between the PANDA nutrition intervention and other similar programs is our emphasis on the 4-A Framework. We propose that focusing on the 4-A Framework for the diabetes menu plan for management helped achieve the desired effect. Providing locally grown or imported ingredients (Food Availability) may have increased feasibility for the participants to adopt the menu plan. Most recipes used affordable ingredients [53] to help overcome the barrier related to food prices. The menu plan was based on culturally acceptable food for diabetic Albertan participants (Acceptable Food) and it was clear and easy to follow. Thus, the 4-A Framework underpinning the PANDA menu plan helped to ensure adequate nutrient intake (Food Adequacy), and improve diet adherence and health outcomes among individuals with T2D. As part of the evaluation of the intervention, participants also completed pre- and post-intervention questionnaires regarding food availability, accessibility and acceptability, not reported here. Another benefit of the education program may have been the emphasis on appropriate portion sizes, as evidenced by the reduction in the number of participants who under-reported dietary intake from baseline to three months.

A strength of this study is that we translated the complex CDA nutrition recommendations, and the serving recommendations suggested in CFG, into a simple and practical menu plan, around which the theory-based education program was built. The menu plan also took into consideration the 4-A Framework. Several outcomes including A1c, lipid profile and anthropometric measurements were measured at the end of the intervention and three months after the final contact. We were able to compare these biological outcomes to nutritional changes using a web-based 24-h recall platform that participants readily used. The study used several strategies to increase retention rate, including reimbursement for parking or public transit expenses and a gift card for $50 of groceries for participating. Telephone and email reminders were also helpful. However, during most trials participants are lost to follow-up. In our study, the attrition rate at three months was 15% (about half that predicted from our pilot study [17]), and at six months 42%. There are various reasons for participants not attending follow-up appointments particularly at six months such as taking holidays, lack of time, conflict with travel and lack of motivation or interest. Repeating the analyses using a per-protocol approach yielded similar results [54]. There are several other limitations of this study. First, the study did not include a control group and participants were self-selected volunteers, which may affect overall motivation. Not having a control group may be a weakness but disadvantages of randomized controlled trials for dietary interventions, particularly those that elicit variable behavior changes in participants, have been noted [55]. A recent meta-analysis found that behavioral interventions consistently result in lowering of BMI and A1c superior to controls in trials with randomization [38]. The majority of the participants were Caucasian; therefore, we cannot generalize the results to all ethnicities. Even though dietary intakes were assessed with a validated internet-based questionnaire, measurement error may occur due to inaccurate reporting by participants. In addition, the platform could not distinguish between home-cooked or restaurant meals, such details as types of oil in salad dressings or homemade vs. canned soup, which may therefore under-estimate shifts in consumption patterns. Therefore, changes in biological variables may more accurately reflect the effectiveness of the program than analysis of dietary intake. In addition, the study did not assess dietary adherence in the follow-up assessment. Medication use can affect the biological outcomes we measured; although no changes in medication were reported, missing data from 14% of participants limited our ability to analyze changes in drug regimens as a potential confounder.

## 5. Conclusions

In summary, the menu plan delivered as part of an education program led to significant improvements in glycemic control, lipid profile, and anthropometric measurements that were sustained over six months. In addition, positive changes in dietary habits, including reduced sodium intake were documented. The PANDA Nutrition Arm was shown to be effective and feasible for improving clinical outcomes in diabetic patients. Further research is warranted to examine its delivery in a community-based model.

## Figures and Tables

**Table 1:** Baseline characteristics of all participants.

Variable	Total Cohort	Men	Women	*p*-Value *
	(*n* = 73)	(*n* = 39)	(*n* = 34)	
**Demographic Variables**				
Age (year ± SD)	59.2 ± 9.7	59.0 ± 10.2	59.5 ± 9.1	0.846
Ethnicity (%)				
White	87.7	84.6	91.2	0.24
Other	12.3	15.4	8.8	
Education (%)				
High school or less	15	15.4	14.7	0.215
More than high school	85	84.6	85.3	
Employment Status (%)				
Working	56.2	56.4	55.9	0.81
Other ^1^	43.8	43.6	44.1	
Annual income (%)				
<$60,000	21.9	15.3	29.4	0.165
>$60,000	78.1	84.7	70.6	
**Diabetes-Related Variables**				
Duration of T2D (year ± SD)	9.1 ± 8.3	10.8 ± 9.6	7.0 ± 5.8	0.049
Diabetes treatment (%)				
Oral medication	74	66.6	82.2	0.438
Diet + exercise	6.8	7.6	5.8	
Insulin	10.9	12.8	8.8	
Combination ^2^	8.2	10.2	5.8	
Additional medication (%)				
Anti-hypertensive drugs	57.5	61.5	52.9	0.459
Lipid-lowering drugs	47.9	53.8	41.1	0.28
A1c (% ± SD)	8.0 ± 1.8	8.3 ± 1.7	7.7 ± 1.9	0.143
Diabetes Self Efficacy Scale score (maximum 10)	7.1 ± 1.5	7.5 ± 1.2	6.6 ± 1.8	0.012
**Anthropometric and Physical Activity Variables**				
Weight (kg ± SD)	96.4 ± 21.0	98.6 ± 20.8	93.8 ± 21.4	0.336
Body mass index (kg/m^2^ ± SD)	32.5 ± 6.8	31.3 ± 6.4	33.8 ± 7.1	0.117
Waist circumference (cm ± SD)	110.8 ± 16.8	112.2 ± 16.5	109.1 ± 17.2	0.336
Fat mass (kg ± SD)	40.0 ± 15.7	36.0 ± 14.4	44.6 ± 16.1	0.019
Fat mass (% ± SD)	40.4 ± 9.1	35.3 ± 7.6	46.2 ± 7.0	<0.001
Fat-free mass (kg ± SD)	56.6 ± 10.5	62.9 ± 9.0	49.3 ± 6.9	<0.001
Fat-free mass (% ± SD)	59.6 ± 9.1	64.7 ± 7.6	53.8 ± 7.0	<0.001
Physical activity (steps/day ± SD)	5535 ± 3491	6722 ± 3829	4330 ± 2375	0.002
**Blood Pressure and Lipid Variables**				
Systolic blood pressure (mmHg ± SD)	128.5 ± 13.5	132.5 ± 15.0	124.0 ± 10.0	0.007
Diastolic blood pressure (mmHg ± SD)	78.6 ± 8.9	80.4 ± 10.2	76.5 ± 6.7	0.066
Total cholesterol (mg/dL ± SD)	328.7 ± 82.7	326.8 ± 77.8	330.9 ± 89.1	0.833
HDL-cholesterol (mg/dL ± SD)	57.6 ± 24.5	58.6 ± 25.5	56.5 ± 23.8	0.724
LDL-cholesterol (mg/dL ± SD)	243.9 ± 80.1	241.8 ± 76.8	246.3 ± 85.1	0.812
Triglycerides (mg/dL ± SD)	135.9 ± 73.5	132.0 ± 58.9	140.4 ± 88.2	0.632
**Nutrient Intake Variables**				
Energy (kcal)	2109 ± 721	2161 ± 598	2046 ± 845	0.494
Energy under-reported (*n* (%)) ^3^	49 (67.1)	29 (74.4)	20 (58.8)	0.803
Energy acceptably reported (*n* (%))	18 (24.7)	9 (23.1)	8 (26.5)	
Energy over-reported (*n* (%))	6 (8.2)	1 (2.6)	5 (14.7)	
Total fat (g)	86 ± 36	87 ± 35	84 ± 37	0.717
Total fat (% of energy)	36 ± 7	35 ± 7	36 ± 6	0.43
Protein (g)	99 ± 30	103 ± 30	94 ± 28	0.183
Protein (% of energy)	19 ± 4	19 ± 3	19 ± 4	0.812
Carbohydrate (g)	238 ± 93	241 ± 64	234 ± 118	0.752
Carbohydrate (% of energy)	45 ± 7	45 ± 6	45 ± 7	0.919
Fibre (g) ^4^	22 ± 7	21 ± 7	21 ± 7	0.888
Added sugar (g)	50 ± 47	43 ± 24	56 ± 63	0.254
Added sugar (% of energy)	9 ± 5	8 ± 4	9 ± 6	0.123
Saturated fat (g)	28 ± 13	28 ± 11	27 ± 15	0.932
Saturated fat (% of energy)	12 ± 3	12 ± 3	11 ± 3	0.583
MUFA (g)	30 ± 13	29 ± 12	30 ± 14	0.889
MUFA (% of energy)	12 ± 3	11 ± 3	12 ± 3	0.105
PUFA (g)	15 ± 7	14 ± 7	15 ± 6	0.619
PUFA (% of energy)	6 ± 2	5 ± 2	7 ± 2	0.36
Sodium (g)	3.36 ± 1.56	3.57 ± 1.50	3.11 ± 1.60	0.217
Sodium density (mg/1000 kcal)	1.6 ± 0.5	1.6 ± 0.5	1.5 ± 0.4	0.284
**Diet Quality and Adherence Variables**				
HEI score (maximum 100)	68.7 ± 8.9	68.1 ± 8.1	69.3 ± 9.8	0.533
PDAQ score (maximum 63)	32.3 ± 11.3	32.9 ± 10.6	31.5 ± 12.1	0.611

Data presented based on the per-protocol analysis; * *p* < 0.05. Student’s unpaired *t*-test for continuous and Χ^2^ test for categorical variables; ^1^ Unemployed or retired; ^2^ Oral medication and insulin; ^3^ Estimated from the Institutes of Medicine method [23] and the Goldberg cut off for acceptable energy intake [25]; ^4^ Dietary fiber only, does not include supplements.

**Table 2:** Changes in biological outcomes at three and six months ^1^.

Outcome Variable	3 Months		6 Months	
	Mean Change	95% CI	Mean Change	95% CI
**Diabetes-related Variables ^2^**				
A1c (%)	−0.7	(−1.0, −0.4) ***	−0.5	(−0.9, −0.1) **
Diabetes Self-Efficacy Scale (score)	0.7	(0.3, 1.0) **	ND	ND
**Anthropometric Variables and Physical Activity**				
Weight (kg)	−1.7	(−2.2, −1.2) ***	−1.4	(−2.1, −0.8) ***
BMI (kg/m^2^)	−0.6	(−0.8, −0.4) ***	−0.5	(−0.7, −0.3) ***
Waist circumference (cm)	−2.4	(−3.0, −1.8) ***	−2.4	(−3.0, −1.8) ***
Fat mass (kg)	−1.2	(−2.0, −0.4) **	ND	ND
Fat mass (%)	−0.8	(−1.5, 0.0)	ND	ND
Fat free mass (kg)	−0.8	(−1.8, 0.1)	ND	ND
Fat free mass (%)	0.8	(0.1, 1.6) *	ND	ND
Physical activity (steps/day)	995	(368, 1623) **	ND	ND
**Blood Pressure and Lipids**				
Systolic blood pressure (mm Hg)	−4.1	(−6.8, −1.3) **	ND	ND
Diastolic blood pressure (mm Hg)	−1.7	(−3.1, −0.4) *	ND	ND
Total cholesterol (mg/dL)	−63.5	(−80.1, −46.9) ***	−86.2	(−107.3, −65.2) ***
HDL-cholesterol (mg/dL)	27.5	(20.2, 34.8) ***	44.6	(37.2, 52.0) ***
LDL-cholesterol (mg/dL)	−88.9	(−105.3, −72.5) ***	−128.3	(−148.5, −108.2) **
Triglycerides (mg/dL)	−10.4	(−23.1, 2.2)	−3.8	(−20.8, 13.2)

ND Not done; Data presented for *n* = 73 based on intention-to-treat analysis with imputed data; Paired *t*-test comparisons for each assessment, * *p* < 0.05; ** *p* < 0.001; *** *p* < 0.0001; ^1^ Men and women were also analyzed separately but trends were similar for both; hence only data for the combined cohort are presented; ^2^ Data are for *n* = 73 participants with missing data imputed.

**Table 3:** Changes in nutrients and food groups, diet quality, perceived dietary adherence at 3 months derived from repeated 24-h recalls.

Nutrient and Diet Score Variables	Mean Change	95% CI
**Nutrient Intake**		
Energy (kcal)	−178	(−304, −51) **
Total Fat (g)	−10.2	(−17.6, −2.7) **
Total Fat (%)	−1.1	(−2.5, 0.4)
Protein (g)	−5.8	(−11.1, −0.4) *
Protein (%)	0.4	(−0.4, 1.3)
Carbohydrate (g)	−11.8	(−27, 3.5)
Carbohydrate (%)	1.9	(−0.2, 3.7)
Fiber (g)	0.0	(1.3, 0.0)
Added sugar (g)	−8.5	(−16, −2.1) *
Added sugar (%)	−0.3	(−1.4, 0.8)
Saturated fat (g)	−3.5	(−6.3, −0.6) *
Saturated fat (%)	−0.4	(−1.2, 0.4)
MUFA (g)	−2.7	(−5.6, 2)
MUFA (%)	0.1	(−0.7, 1.0)
PUFA (g)	0.1	(−1.6, 1.8)
PUFA (%)	0.8	(0.1, 1.4) *
Sodium (g)	−0.57	(−0.87, −0.28) ***
Sodium density (mg/kcal)	−0.14	(−0.26, −0.03) *
**Diet quality and adherence**		
**Health Eating Index**		
Health Eating Index score (maximum 100)	2.1	(0.1, 4.1) *
Total fruits and vegetables (maximum 10)	0.5	(0.1, 0.9) *
Whole fruits (maximum 5)	0.4	(0.1, 0.7) *
Dark green/orange vegetables (maximum 5)	−0.1	(−0.4, 0.3)
Total grains (maximum 5)	−0.3	(−0.6, −0.2) *
Whole grains (maximum 5)	0.3	(−0.1, 0.7)
Dairy (maximum 10)	−0.2	(−0.6, 0.3)
Meat/beans (maximum 10)	0.2	(−0.3, 0.6)
Unsaturated fat (maximum 10)	−0.1	(−0.6, 0.4)
Saturated fat (maximum 10)	0.9	(0.1, 1.7) *
Sodium (maximum 10)	1.1	(0.4, 1.7) **
Other (maximum 20) ^1^	−0.1	(−1.3,1.1)
**Perceived Dietary Adherence**		
Perceived dietary adherence score (maximum 63)	8.5	(6.1, 10.8) ***

Data presented based on intention-to-treat analysis with imputed data; Paired *t*-test comparisons, * *p* < 0.05; ** *p* < 0.001; *** *p* < 0.0001; ^1^ For calories from solid fats, alcohol and added sugars, a higher score indicates lower consumption; likewise for saturated fat and sodium scores, a higher score indicates lower consumption.

**Table 4:** Unadjusted and adjusted multiple linear regressions examining variables as predictors of A1c change after the PANDA intervention.

Model	Variables	Change in A1c (%) Per Unit Change Invariable of Interest	95% CI
Model 1 *	Increase in PA (100 Steps)	−0.002	−0.040 to 0.000
	Increase in HDL-C (10 mg/dL)	−0.054	−0.081 to −0.027
	Increase in HEI (1 unit)	−0.018	−0.038 to 0.001
	Decrease in BMI (1 kg/m^2^)	−0.081	−0.030 to 0.019
	Decrease in total calories (10 kcal)	0.07	−0.040 to 0.180
Model 2 **	Increase in PA (100 Steps)	−0.004	−0.011 to 0.002
	Increase in HDL-C (10 mg/dL)	−0.021	−0.041 to 0.001
	Increase in HEI (1 unit)	−0.019	−0.029 to 0.002
	Decrease in BMI (1 kg/m^2^)	−0.112	−0.194 to −0.029
	Decrease in total calories (10 kcal)	0.033	−0.048 to 0.114

* unadjusted; ** adjusted for age, gender, baseline A1c, baseline BMI, baseline HEI, and baseline physical activities.

**Table 5:** Unadjusted and adjusted multiple linear regressions examining variables as predictors of HEI change after the PANDA intervention.

Model	Variables	Change in HEI (Score) Per Unit Change in Variable of Interest	95% CI
Model 3 *	Decrease in total calories (10 kcal)	2.71	−0. 42 to 5.83
	Decrease in total fat (1 g)	−0.021	−0.205 to 0.164
	Decrease saturated fat (1 g)	−0.111	−0.186 to −0.035
	Decrease total sugar (1 g)	−0.002	−0.016 to 0.012
	Decrease sodium intake (10 mg)	−0.60	−1.69 to 0.50
Model 4 **	Decrease in total calories (10 kcal)	2.66	−0.60 to 5.91
	Decrease total fat (1 g)	−0.018	−0.216 to 0.181
	Decrease saturated fat (1 g)	−0.117	−0.195 to −0.039
	Decrease total sugar (1 g)	−0.002	−0.016 to 0.012
	Decrease sodium intake (10 mg)	−0.53	−1.68 to 0.62

Variables shown in text are significant predictors (*p* < 0.05); * unadjusted; ** adjusted for age, gender, baseline A1c, and HEI baseline.

## Supplementary Materials

The following are available online at www.mdpi.com/2227-9032/4/4/73/s1, Table S1: A one-day sample menu.
