# Supplementary material for: Effectiveness of a Lifestyle Intervention in Patients with Type 2 Diabetes: The Physical Activity and Nutrition for Diabetes in Alberta (PANDA) Trial
Source: Healthcare (Basel). 2016 Sep 27;4(4):73. doi: 10.3390/healthcare4040073 (PMC5198115; doi:10.3390/healthcare4040073)
Supplement: Supplementary file 1 [file healthcare-04-00073-s001.pdf]

Supplementary Materials: Effectiveness of a Lifestyle Intervention in Patients with Type 2 Diabetes: The Physical Activity and Nutrition for Diabetes in Alberta (PANDA) Trial

Ghada Asaad, Diana C. Soria-Contreras, Rhonda C. Bell and Catherine B. Chan

Table S1. A one-day sample menu.

Table S1 A. Week 1, Day 1.

Meal	Ingredients Per Serving	Canada's Food Guide Servings
Breakfast<br>Breakfast Parfait (see recipe, page 25 [1])	½ cup frozen berries<br>¼ cup low-fat granola<br>¾ cup low-fat yogurt<br>½ cup apple juice<br>1 cup coffee/tea<br>2 tbsp 1% milk (optional)<br>1 tsp sugar (optional)	2 Vegetables and Fruit<br>1 Grain Products<br>1 Milk and Alternatives
Morning Snack<br>Oat Bran Applesauce Muffins with Roasted Almonds (see recipe, page 26 [1])	1 oat bran applesauce muffin<br>1 tsp margarine<br>1 tbsp roasted almonds	1 Grain Products<br>¼ Meat and Alternatives<br>1 Oils and Fats
Lunch<br>Tuna Caesar Sandwich (see recipe, page 27 [1])	2.5 oz white tuna<br>1 tbsp artichoke hearts<br>1 tbsp onion<br>1 tbsp fat-free mayonnaise<br>¾ tbsp Parmesan cheese<br>¼ cup each cucumber and tomato<br>½ cup lettuce<br>2 slices whole wheat toast<br>1 cup 1% milk<br>½ cup canned peaches (in water)	2½ Vegetables and Fruit<br>2 Grain Products<br>1 Meat and Alternatives<br>1 Milk and Alternatives<br>1 Oils and Fats
Afternoon Snack<br>Hummus & Crackers (see recipe, page 28 [1])	¼ cup hummus<br>6 Melba toast<br>¼ cup chopped tomatoes	½ Vegetables and Fruit<br>1½ Grain Products<br>½ Meat and Alternatives
Dinner<br>Pork Tenderloin with Roasted Potatoes (see recipe, page 29 [1])	4 oz pork tenderloin<br>⅔ cup roasted potatoes<br>½ cup green beans<br>1 small whole grain dinner roll<br>1 tsp margarine<br>½ cup 1% milk	2 Vegetables and Fruit<br>1 Grain Products<br>1½ Meat & Alternatives<br>½ Milk and Alternatives<br>1 Oils and Fats
Evening Snack<br>Cinnamon Raisin Toast	1 slice toasted raisin bread<br>1 tsp margarine<br>1 tsp cinnamon<br>½ cup 1% milk	1 Grain Products<br>1 Milk and Alternatives<br>1 Oils and Fats
	Total serving	7½ Grain Products<br>3¼ Meat and Alternatives<br>3½ Milk and Alternatives<br>4 Oils and Fats

Table S1 B. Good to Know.

**Nutrition Fact of the Day**	**Adjusting Today's Menu**
	To cut about 200 calories
Calories: 1848	• Have only ½ cup of yogurt at breakfast (saves 50 kcal)
Fat: 49 g	• Omit margarine at morning snack and dinner (saves 70 kcal)
Saturated fat: 13 g	• Make an open-faced sandwich for lunch (saves 70 kcal)
Carbohydrate: 262 g	To add about 200 calories
Fibre: 28 g	• Have 2 tbsp almonds at morning snack (adds 50 kcal)
Protein: 100 g	• Drink 1 cup of milk with your dinner (adds 50 kcal)
	Have 2 slices of toast at evening snack (adds 100 kcal)

Reference

1. Chan, C.B.; Bell, R.C. *Pure Prairie Eating Plan—Fresh Food, Practical Menus and a Healthy Lifestyle*; Division of Human Nutrition, University of Alberta: Edmonton, AB, Canada, 2013.

© 2016 by the authors. Submitted for possible open access publication under the terms and conditions of the Creative Commons Attribution (CC-BY) license (http://creativecommons.org/licenses/by/4.0/).
